# RESEARCHING THE AGING POPULATION IN STATE PRISONS: ETHICAL CONSIDERATIONS AND IRB REQUIREMENTS

**DOI:** 10.1093/geroni/igae098.1787

**Published:** 2024-12-31

**Authors:** Rodlescia Sneed, Elaina Reese

**Affiliations:** Wayne State University, Detroit, Michigan, United States; Wayne State University, Detroit, Michigan, United States

## Abstract

The growing number of older adults within state prisons presents unique challenges and opportunities for gerontological research. To conduct research with this population, however, scholars must navigate a range of ethical issues unique to studying this vulnerable group. The purpose of this presentation is to delve into the ethical considerations and Institutional Review Board (IRB) requirements crucial for conducting research with this vulnerable population. Using examples from our current multisite research study on chronic disease self-management among incarcerated older adults, we will describe how we navigated special considerations related to correctional system research processes, power imbalance, coercion, informed consent, participant selection, participant incentives, and data protection. Understanding how to effectively navigate ethical issues and the IRB process is crucial for conducting research to improve the well-being of incarcerated older adults. This presentation will provide researchers with valuable information useful for navigating the ethical landscape to conduct research that improves the well-being of the aging prison population and informs best practices within correctional healthcare.

